# Exome Sequencing Identifies *DLG1* as a Novel Gene for Potential Susceptibility to Crohn's Disease in a Chinese Family Study

**DOI:** 10.1371/journal.pone.0099807

**Published:** 2014-06-17

**Authors:** Shufang Xu, Feng Zhou, Jinsheng Tao, Lu Song, Siew Chien NG, Xiaobing Wang, Liping Chen, Fengming Yi, Zhihua Ran, Rui Zhou, Bing Xia

**Affiliations:** 1 Department of Gastroenterology, Zhongnan Hospital of Wuhan University School of Medicine, Wuhan, People’s Republic of China; 2 Hubei Clinical Center and Key Laboratory for Intestinal and Colorectal Diseases, and Hubei Key Laboratory of Immune Related Diseases, Wuhan, People’s Republic of China; 3 BGI-Shenzhen, Bei Shan Industrial Zone, Yantian District, Shenzhen, People’s Republic of China; 4 Institute of Digestive Disease, Department of Medicine and Therapeutics, Li Ka Shing Institute of Health Sciences, The Chinese University of Hong Kong, Hong Kong, People’s Republic of China; 5 Department of Gastroenterology, Renji Hospital, Shanghai Institute of Digestive Disease, Shanghai Jiao Tong University School of Medicine, Shanghai, People’s Republic of China; Northwestern University Feinberg School of Medicine, United States of America

## Abstract

**Background:**

Genetic variants make some contributions to inflammatory bowel disease (IBD), including Crohn’s disease (CD) and ulcerative colitis (UC). More than 100 susceptibility loci were identified in Western IBD studies, but susceptibility gene has not been found in Chinese IBD patients till now. Sequencing of individuals with an IBD family history is a powerful approach toward our understanding of the genetics and pathogenesis of IBD. The aim of this study, which focuses on a Han Chinese CD family, is to identify high-risk variants and potentially novel loci using whole exome sequencing technique.

**Methods:**

Exome sequence data from 4 individuals belonging to a same family were analyzed using bioinformatics methods to narrow down the variants associated with CD. The potential risk genes were further analyzed by genotyping and Sanger sequencing in family members, additional 401 healthy controls (HC), 278 sporadic CD patients, 123 UC cases, a pair of monozygotic CD twins and another Chinese CD family.

**Results:**

From the CD family in which the father and daughter were affected, we identified a novel single nucleotide variant (SNV) c.374T>C (p.I125T) in exon 4 of discs large homolog 1 (*DLG1*), a gene has been reported to play mutiple roles in cell proliferation, T cell polarity and T cell receptor signaling. After genotyping among case and controls, a PLINK analysis showed the variant was of significance (*P*<0.05). 4 CD patients of the other Chinese family bore another non-synonymous variant c.833G>A (p.R278Q) in exon 9 of *DLG1.*

**Conclusions:**

We have discovered novel genetic variants in the coding regions of *DLG1* gene, the results support that *DLG1* is a novel potential susceptibility gene for CD in Chinese patients.

## Introduction

Crohn’s disease (CD) and ulcerative colitis (UC) are classified as chronic, idiopathic inflammatory bowel diseases (IBD) [1], [2]. Familial aggregation, high concordance in twins and a higher prevalence of the disease in a certain ethnic population imply a strong genetic influence on the risk of disease development [3], [4]. Identifying the genetic loci or rare detrimental mutations in different populations or families with the disease will help elucidate the pathogenesis of these complex traits and facilitate the development of more targeted therapy.

It is now widely recognized that common variants shown in GWAS can explain only relatively modest proportions of risk for diseases. Numerous functional and deleterious variants in the population are at frequencies of 0.5 to 5% that are too low to be detected by GWAS [5], [6]. As predisposing variants will present at a much higher frequency in the affected relatives of an index case, family studies may facilitate the detection of the ‘missing heritability’ not identified by GWAS [7]. Exome sequencing, which is a technique that focuses on the protein-coding portion of the genome, is not limited by the detailed and complete pedigree data that are necessary for classical linkage analysis and can be performed on only a few patients for the detection of causal mutations [8], [9].

Researchers have successfully identified a causal hemizygous mutation in the XIAP gene [10] and novel compound heterozygous mutations in interleukin-10 receptor 1 (IL-10R1) [11], using exome sequencing in children presenting with very early-onset and intractable IBD. The sequencing of eight pediatric IBD patients’ exomes revealed various profiles of specific variants with a limited number in each case [12].

Numerous candidate genes for Western IBD patients have been shown, but causality for specific variants in Chinese IBD patients is largely absent. In this study, we applied whole exome sequencing to 4 individuals belonging to a same family (Family A) to discover novel deleterious genetic variants associated with IBD and then validated these findings in other 10 family members of Family A, 401 healthy controls (HC), 278 subjects with sporadic CD, 123 subjects with UC, a pair of monozygotic twins and another Han Chinese CD family (Family B).

## Materials and Methods

### Patients and Controls

The familial patients included in this study were selected from the Hubei Clinical Center & Key Lab of Intestinal & Colorectal Diseases. Written informed consent was obtained from all subjects and the next of kin on behalf of the children enrolled in the study. This study was approved by the ethics committee of the Zhongnan Hospital of Wuhan University as part of the human subjects’ protocol to study the genetics of IBD in humans. The CD patients and HC were all unrelated subjects of Chinese descent and born to non-consanguineous parents. The ancestry of the patients and control individuals was assessed by self-report and appearance. Phenotypic data were acquired from a review of medical records, phone interviews and photographs. A combination of symptom assessment, laboratory and radiological examinations and endoscopy with histology was applied to make the diagnosis.

For whole exome sequencing, we selected a Han Chinese family (Family A) including a daughter and a father both affected with CD from Hubei province. In this family, the father is the proband, the proband’s unaffected mother and wife were taken as exome sequencing controls. The father, who was diagnosed with CD at the age of 31 years in 1999 with terminal ileitis and proctitis, was treated with oral prednisolone and aminosalicylic acid (5-ASA). Small intestine computed tomography enterography (CTE) showed a thickened ileum wall in 2012.

**Figure 1:**
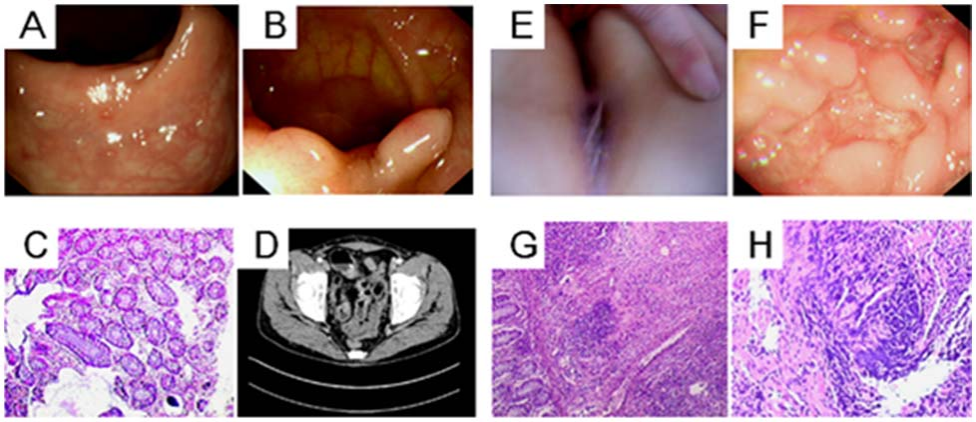
Clinical characteristics of two patients in Chinese CD family A. The index patient in the family, the father (Panels A to D) had evidence of mucous membrane granulation, polypoid proliferation and hyperemia in his colonoscopy, as shown in Panels A and B. Panel C shows the patient’s pathological findings of chronic intestinal inflammation. Panel D shows the thickening of the ileum wall by small intestine computed tomography enterography (CTE). The daughter in the family is another Patient (Panels E to H). Panel E shows her anal fistula at disease onset. Endoscopy showed intestinal poly-ulcers in Panel F. A biopsy showed non-specific granulomatous inflammation, as shown in Panel G, and the higher magnification of the pathology shown in Panel H reveals negative acid-fast staining granulomas. All of the images were collected in March 2012 in Zhongnan Hospital of Wuhan University.

The affected daughter developed CD at the age of 16 years in 2012, with high fever, diarrhea, oral ulcers and an anal fistula. Endoscopy showed upper digestive tract ulcers, aphthous ulcers at the ileocecal junction and colitis involving the rectum, sigmoid colon, descending colon and transverse colon. A biopsy showed non-specific granulomatous inflammation and staining was negative for acid-fast bacilli. She was finally diagnosed with CD and was treated with an intravenous injection of corticosteroids, 5-ASA, immunosuppressants and infliximab for severe refractory disease. She is now in remission with azathioprine and 5-ASA (the supporting data are provided in Fig. 1).

An additional 10 healthy members’ blood DNA samples from family A were also taken as Sanger sequencing controls to validate the co-segregation of the mutations in the CD family.

In addition, 278 sporadic CD and 123 sporadic UC patients were enrolled from the Inflammatory Bowel Disease Center of Zhongnan Hospital of Wuhan University (131 CD, 76 UC), Renji Hospital of Shanghai Jiaotong University School of Medicine (40 CD) and the Institute of Digestive Disease of The Chinese University of Hong Kong (107 CD, 47 UC) from January 2001 to December 2012; 401 HC were from Wuhan, China (Table 1).

**Table 1:** Characteristics of 401 sporadic inflammatory bowel disease (*IBD*) patients and 401 healthy controls.

Index	CD 278	UC 123	HC
Male (n = )	176	72	239
Female (n = )	102	51	162
Average age (years)	32.25±13.38	35.40±10.63	36.42±12.42

Moreover, we collected 25 young and intractable CD cases (Table 2), including a pair of monozygotic twins (Patient ID in Table 2: 24 and 25) and 23 cases selected from 131 sporadic CD patients of Hubei province. Another CD family (family B) was also from Wuhan city.

**Table 2:** Data of 25 young and intractable CD patients.

PatientID	Age atdiagnosis(year)	Sex	Diseaselocation	Treatment					
				Diagnosticanti-TB	Aminosalicylates	Corticosteroids	Immunosuppressiveagentes	Biologicalagentes	Surgery
1	25	Female	Terminal ileum	NO	YES	NO	NO	NO	YES
2	15	Male	Terminal ileum	NO	YES	YES	NO	YES	NO
3	24	Male	Terminal ileum	NO	YES	NO	YES	NO	NO
4	34	Male	Terminal ileum and ascendingcolon	YES	YES	NO	YES	NO	YES
5	16	Male	Terminal ileumand sigmoid colon	YES	YES	YES	YES	YES	NO
6	14	Male	Terminal ileum	YES	YES	NO	YES	YES	NO
7	19	Female	Terminal ileum and right sidedcolon	NO	YES	YES	YES	YES	YES
8	17	Male	Terminal ileum and descendingcolon	YES	YES	YES	YES	NO	NO
9	21	Male	Small intestine	NO	YES	YES	YES	NO	NO
10	20	Male	Terminal ileum and right sidedcolon	YES	YES	YES	YES	YES	YES
11	24	Female	Terminal ileum	NO	YES	NO	NO	YES	NO
12	21	Male	Terminal ileum	YES	YES	YES	YES	YES	NO
13	22	Female	Terminal ileumand sigmoid colon	YES	YES	YES	YES	YES	NO
14	23	Male	Terminal ileum and right sidedcolon	NO	YES	YES	YES	YES	NO
15	23	Male	Small intestine	NO	YES	YES	YES	YES	NO
16	17	Male	Terminal ileum and right sidedcolon	NO	YES	YES	YES	YES	NO
17	13	Female	Terminal ileum and ascendingcolon	YES	YES	YES	YES	YES	YES
18	24	Female	Terminal ileum	NO	YES	YES	YES	YES	NO
19	25	Male	Terminal ileum and ascendingcolon	NO	YES	YES	YES	YES	YES
20	11	Male	Terminal ileum and right sidedcolon	YES	YES	YES	YES	YES	YES
21	21	Female	Terminal ileum and ascendingcolon	YES	YES	YES	YES	NO	NO
22	14	Female	Terminal ileum and right sidedcolon	NO	YES	YES	YES	NO	YES
23	11	Male	Terminal ileum and right sidedcolon	YES	YES	YES	YES	YES	YES
24	26	Male	Terminal ileum	NO	YES	YES	YES	NO	YES
25	29	Male	Terminal ileum	NO	YES	YES	YES	YES	YES

TB: tuberculosis.

### DNA Extraction

Genomic DNA was extracted from EDTA-anticoagulated peripheral venous blood samples using a QIAamp DNA Blood Midi Kit (Qiagen, Germany) according to the manufacturer’s instructions.

### Whole Exome Sequencing and Variant Detection

Using an E210 ultrasonicator (Covaris, MA, USA), the genomic DNA samples were randomly fragmented into 250–300 bp fragments and subjected to library preparation according to NimbleGen’s standard protocol. Target region enrichment was performed for the shotgun libraries using the NimbleGen SeqCap EZ custom design kit (NimbleGen, Madison, WI, USA), which consisted of SeqCap EZ Human Exome Library v2.0 and a continuous region covering the MHC genes. The enriched shotgun libraries were sequenced using the Hiseq2000 platform, and 90-bp paired-end reads were generated. Raw image data and base calling were processed by Illumina Pipeline software version 1.7 with the default parameters. Quality control for the reads was performed by discarding adaptor-containing reads and low-quality reads. For SNP calling, SOAP aligner [13] was used to align the reads to the human reference genome (hg19), and SOAP snp [14] was then used to assemble the consensus sequence and call SNPs. As another quality control, low-quality SNPs satisfying one of the four following criteria were discarded: (i) genotype quality<20; (ii) total reads covering the variant site<4; (iii) estimated copy number >2; (iv) distance from the nearest SNP<5 bp (except for SNPs present in dbSNP). For indel calling, high-quality reads were aligned to the human reference genome using BWA (version 0.5.9-r16) [15]. GATK Indel Realigner was used to realign reads around insertion/deletion sites, and then small indels were called using the IndelGenotyperV2 tool from GATK (version v1.0.4705) [16], [17]. Indels were called as heterozygous and homozygous if indel-supporting reads consisted of 30–70% and >70% of the total reads, respectively. SNP and indel detection was performed only for the targeted regions and flanking regions within 200 bp of the targeted regions.

### Variant Annotation and Prioritization

The detected variants were annotated based on four databases, including NCBI CCDS, RefSeq, Ensembl and Encode (http://genome.ucsc.edu/ENCODE/). Exclusion steps were taken to help identify candidate mutations. Variants falling within intergenic, intronic and untranslated regions and synonymous substitutions were excluded; variants documented in 4 public genetic variant databases, including dbSNP132, 1000 Genomes, HapMap and YH (http://yh.genomics.org.cn/), with an allele frequency >0.5% (except for YH) were rejected; and variants shared by 2 exome sequenced cases and absent from 2 exome sequenced controls were kept. Additionally, we used the following criteria to evaluate and prioritize the candidate genes: (i) SIFT (http://sift.bii.a-star.edu.sg/), MutationTaster (http://www.mutationtaster.org/), PolyPhen-2 (http://genetics.bwh.harvard.edu/pph2/index.shtml) and PMut (http://mmb.pcb.ub.es/PMut/) were used to predict whether single amino acid changes in genes would alter the protein function; (ii) the conservation of candidate mutations was analyzed by evaluating the GERP score (http://snp.gs.washington.edu/SeattleSeqAnnotation137/); (iii) the candidate mutations’ total frequency of occurrence in the EVS (http://evs.gs.washington.edu/EVS/) and the BGI in-house database was analyzed and (iiii) the tissue distributions and functions were analyzed using the online tools BioGPS (http://biogps.org/), Entrez Gene (http://www.ncbi.nlm.nih.gov/gene) and Proteinatlas (http://www.proteinatlas.org/).

As a final step, we compared and prioritized the remaining candidate genes using 4 internet tools: GeneDistiller2 (http://www.genedistiller.org/), SUSPECTS (http://www.genetics.med.ed.ac.uk/suspects/index.shtml), ToppGene (http://toppgene.cchmc.org/) and Endeavour (http://www.esat.kuleuven.be/endeavour). To prioritize the SNVs and genes, we chose 20 reported CD susceptibility genes (*NOD2*, *ATG16L1*, *STAT3*, *IL23R*, *IL10R2*, *IL10R1*, *JAK2*, *ICOSLG*, *CDKAL1*, *MST1*, *PTGER4*, *IRGM*, *TNFSF15*, *ZNF365*, *NKX2-3*, *PTPN2*, *PTPN22*, *IL12B*, *XIAP* and *ITLN1*) as the training set.

### Validation Phase

All shared SNVs of the two affected individuals were verified for all members acquired from family A to detect co-segregation, by direct polymerase chain reaction (PCR) amplification followed by Sanger sequencing (PCR primers are listed in Table 3, Invitrogen). The sequencing reactions were conducted on an ABI 3730XL DNA Analyzer.

**Table 3:** The PCR primers of 22 candidate SNPs by Sanger Sequencing.

SNP_ID	Gene	F-primer sequences (5′-3′)	R-primer sequences (5′-3′)
chr17_55183450	*AKAP1*	TCAGAGTCCTCGGGCATT	CTGCTACATACTCTTCCTCC
chr20_30232655	*COX4I2*	ACAGTCCTTGGGGTCTAA	CCACTGCTTCTTCTCATAG
chr9_110249480	*KLF4*	AGTCCCGCCGCTCCATTA	TCTTTGGCTTGGGCTCCT
chr1_26368197	*SLC30A2*	ACTGCCTTATTCTGAACTGT	GAAGCATAATCCTCACCC
chr7_73279329	*WBSCR28*	GAGAATCGCCCGAAACC	CCAGGCACTGAGCAAGG
chr9_125582872	*PDCL*	GATTCTTGTTGTGCCTCAG	TTCCTGGTGAACTGACTGC
chr6_90418252	*MDN1*	AACCTCTTCCCCATCAT	TCCAACACCCCACAACT
chr20_420894	*TBC1D20*	GACCTGACACCTGCCTTTC	ACCCAGCATTTCCCAACT
chr9_2717768	*KCNV2*	CCACAGCCAGGAGGAAA	CTCGTAGTCGTCGCACA
chr16_20492206	*ACSM2A*	CAGGGCAGGGGATTTAG	TTGCTGGATCGTATGGTAGTT
chr15_41275952	*INO80*	AGCCAAAGCAGCCTCAAC	GGAATCAGGACCTTACCC
chr6_168366533	*MLLT4*	GAAGCAGGAGGCTGAGAA	TTGAGGTAGGAGGCGTTT
chr3_196921405	*DLG1*	GGTAAGAAATGAGCAATCAATATTCAG	GGGCGAACCTACATGAAAGAATA
chr1_1470881	*TMEM240*	GACGCCTCCGAGAACTACTTTG	ACAGCTTGGGCAGCCAGGTC
chr16_16170185	*ABCC1*	AACCCGTGGCTGATGTC	TGTCCAAGGCTGCTGTA
chr15_39885853	*THBS1*	TGGGTGCTGAGGATGTC	TGGTGATGCTGGGAACT
chr10_116225553	*ABLIM1*	TTCCTTGGCAGTGTTTG	GGAAATGTTTAGTCGTTGA
chr7_98602860	*TRRAP*	TTTCCCGTGACAGTTCG	CTCTTGGTGGTCTCCTTT
chr6_152536152	*SYNE1*	TTGGCTTTTCGCTATTC	ACCTTGACTGCGGACTT
chr14_21860964	*CHD8*	GCCCAAGGTAACAAACAG	CCAGGAGTCAATGAGGGA
chr7_99160120	*ZNF655*	TATGGGCTTTATTCCGTAG	CGGAGAAGACGATGTGAA
chr7_87051466	*ABCB4*	ATCCAAGTGGGCGTTTT	TGAATGTCTGCTGAGGG

Genotyping was conducted by the MassARRAY (MALDI-TOF MS) method using the SEQUENOM System (Sequenom, Inc.) to screen the candidate genes in an additional 401 HC individuals (278 sporadic CD patients and 123 UC cases), and the data were analyzed using TYPER 4.0 software. The primer sequences for genotyping were designed and synthesized using Primer 5.0 software (PCR primer sequences are listed in Table 4, and the primers were synthesized by Invitrogen). To further study the genes (*DLG1* and *PDCL*) that we identified through the series of steps listed above, we applied PCR amplification followed by Sanger sequencing to examine all of the exons of *DLG1* and *PDCL* in 25 young and intractable CD cases (the PCR primer sequences are listed in Table 5).

**Table 4:** The primers of the 22 candidate SNPs for the MassARRAY method.

SNP_ID	Gene	F-primer sequences (5′-3′)	R-primer sequences (5′-3′)
chr17_55183450	*AKAP1*	ACGTTGGATGAGAGGGCAAGAGAGACAGGT	ACGTTGGATGACAGAGCTTCTTCAAGCACC
chr20_30232655	*COX4I2*	ACGTTGGATGAGCGCATGCTGGACATGAAG	ACGTTGGATGCTGCTTCTTCTCATAGTCCC
chr9_110249480	*KLF4*	ACGTTGGATGTCTTTGGCTTGGGCTCCTCT	ACGTTGGATGATGATGCTCACCCCACCT
chr1_26368197	*SLC30A2*	ACGTTGGATGAACCTTGACCATCCTGAGAG	ACGTTGGATGAAGAGCAAAAAGGGAGCCAC
chr7_73279329	*WBSCR28*	ACGTTGGATGTGATGGCTGACGGTTGTCTC	ACGTTGGATGGGAGCAGGAAATTATAGAGG
chr9_125582872	*PDCL*	ACGTTGGATGTGACTCTGAAGGAGTTTGCC	ACGTTGGATGATTCGCTGCTTCCGGTACTG
chr6_90418252	*MDN1*	ACGTTGGATGTTTGATGGACTTTGACCCAC	ACGTTGGATGTGCAGCTGATTCTAAAAGGG
chr20_420894	*TBC1D20*	ACGTTGGATGATGGGTGATGGTGAACCCAG	ACGTTGGATGACCCACTGATGCCGATTTAC
chr9_2717768	*KCNV2*	ACGTTGGATGAGCCATGCTCAAACAGAGTG	ACGTTGGATGCCTCATTCTCCGTCGTGTTC
chr16_20492206	*ACSM2A*	ACGTTGGATGGGTAGAGAATGCACTGATGG	ACGTTGGATGACGGGGTCTGGGCTGCTGAT
chr15_41275952	*INO80*	ACGTTGGATGACAACCAAACCAGTGCTGGG	ACGTTGGATGGTCTCAGATACCGTGAATGG
chr6_168366533	*MLLT4*	ACGTTGGATGAGACAGCACGACGAGGCGG	ACGTTGGATGTAGTCCCGGGGAAGCGGAG
chr3_196921405	*DLG1*	ACGTTGGATGGAACCAATTCTGGACCTATC	ACGTTGGATGGGATGAAGATACACCTCCTC
chr1_1470881	*TMEM240*	ACGTTGGATGAGCCGCCTGACCGCCCCTGT	ACGTTGGATGTGCACAGCTTGGGCAGCCAG
chr16_16170185	*ABCC1*	ACGTTGGATGTGTCCCTGACATGTCTCTGT	ACGTTGGATGTGAATGTGGCATTCCTCACG
chr15_39885853	*THBS1*	ACGTTGGATGTGGCGAGCACCTGCGGAAC	ACGTTGGATGTCCAGGGCTTTGCTTCTTAC
chr10_116225553	*ABLIM1*	ACGTTGGATGTGTACACAGGGGAGTTGATG	ACGTTGGATGAGGATGTTCGGGATCGGATG
chr7_98602860	*TRRAP*	ACGTTGGATGTGCAACACACGCTCCTCTC	ACGTTGGATGTGGCAAGATCTACCCATACC
chr6_152536152	*SYNE1*	ACGTTGGATGCTTCCTTCTAGGGACAGATG	ACGTTGGATGGGTAACCTATATCCAAGCTC
chr14_21860964	*CHD8*	ACGTTGGATGCACAGCTAGTACTCAGACTC	ACGTTGGATGCGAGGTCAATACGGTTTATC
chr7_99160120	*ZNF655*	ACGTTGGATGGATAAACCGAATAATAAGG	ACGTTGGATGACCTCTACAGAGAAGTGATG
chr7_87051466	*ABCB4*	ACGTTGGATGGCAGAAGTGCAACATATTCTC	ACGTTGGATGACCTACCTGAAGGAAGAAAG

**Table 5:** The PCR primers of all exons of *DLG1* and *PDCL.*

Primer ID	F-primer sequences (5′-3′)	R-primer sequences (5′-3′)	Annealing temperature (°C)
***DLG1***			
Exon 1	CCGACTTCTGTCTGTTCTT	GGACCGTGCTGTCTCAT	54
Exon 2	CTCCTCCGTTTTCTAATG	GTTACCGAATGCCTCAG	51.5
Exon 3	GTTAAGTAGTTTGCCTGAACTTGTAGC	CAGATGAAGCCTTGTTGAGGTCT	62
Exon 4	GGTAAGAAATGAGCAATCAATATTCAG	GGGCGAACCTACATGAAAGAATA	55.5
Exon 5	TTTATCTTTATGGCACAGC	AAATGGCAAATCCTGACT	51.5
Exon 6	TTCTGTTTGGTGCTGGAG	GGTCTTCGCATTTGTATC	55.5
Exon 7	CAGAGAAGGATCGGAGGTTGA	GTAAATGGAAACTCTTGGGACTATC	58.8
Exon 8	CCTCCAGAACAAGTCCA	GTATTTATCCCTTATCCAGTC	51.5
Exon 9	TGTTCCTTTTGCTGGCCCTT	ATGACTGCACCACTGGACTC	63
Exon 10	TTCGTAACTCTAGGAGCAGCTGT	CTGTGCATACAAGCCCTCAAC	61
Exon 11	AGACTGGGAGAATAGGAGG	TCACTAATGGCATCACAAC	55.5
Exon 12	TTGAGACTAACCTGGGCAACAT	AAGGACAATTTACCAAGCCTCAA C	61
Exon 13	CTTCTAAGTAGGGGCAGTG	AATAGGTCCAGTGAAAATAAC	54
Exon 14	CAGTAGGCGTGAGAATGTGGC	GCCTGGGCAGTAAGAGTGGA	63
Exon 15	GATTACTGCTGTCTGATGC	GCCTCCTTTGCTACTATG	55.5
Exon 16	TCAATATAACTTACCATTGGATTACAATC	AGTACTATTACCTGTAGTTGCCATGCT	57
Exon 17	TTAAACTCAGAAATGGTGCCTCA	GGTCTGTGAAATGGGTGCTTG	61
Exon 18	AGGTATAAATGAACTATGCTGTCTGAA	CCTTGAAGACAATTAGCAACCTG	58.8
Exon 19	AGTTTGTCCCCTTTGCC	TCAGAATCCCTCCACCC	55.5
Exon 20	AAATAAAGGAGTAGCACATAGC	GAAAGAAGTGGGATAAACAG	54
Exon 21	CATCTTTGGTTGATGGTAGAGTGAG	AGAAAGGACAATAATATGGAGGATG	58.8
Exon 22	ATCCATCCTCCATATTATTGTCCT	ACCCGGCCCTTATCTCCT	57
Exon 23	TTTCATTTCCTATCTAAAGTTTGCTG	ATGGTTCTGCCTCACATTCTGT	57
Exon 24	TGTGTCATCTCTCCTTTGCCA	GAGCCGAGTCATACCATTGC	62
Exon 25	CTATGGGATTGTACCCAGTTTCC	GGTCAGGCCATTCCATCTTC	57
***PDCL***			
Exon 1	TGTCCTGGAAATTGTAGGATCTCA	GACTAGGTTACCTCTGAAAGTGGGA	60.5
Exon 2	ATGTTGGGCATTAGCTTGGC	TTTGACAGGGCTCTATGATTTCTC	60.5
Exon 3-1	TCAAGTGATCCGCTCGTCT	AGCTTCAAGGTCCACAGCA	60.5
Exon 3-2	GCCAGCAGTCAGTTCACCAG	TTTGACAGGGCTCTATGATTTCTC	60.5

SPSS17.0 statistical software was used for statistical analysis, the measurement data were expressed as means +/− standard deviation (SD). PLINK was performed on analysis of genotype data. P values<0.05 were considered as significant.

## Results

### Whole Exome Sequencing of the CD Family

Whole exome sequencing was performed on DNA extracted from the peripheral blood of 4 members of Family A using next-generation sequencing technology. As shown in Table 6, we obtained at least 88.5 million reads that mapped to the target region for each exome, more than 98.5% of the target region was covered and the mean depth of the target region was 128.64×, 148.90×, 202.26× and 158.25×. The summary statistics of the total quality-passing SNPs and indels are all listed in Table 6.

**Table 6:** Summary of original exome sequencing data of four familial individuals.

Exome Capture Statistics	Daughter	Father	Grandmother	Mother
Target region (bp)	48959543	49062223	48959543	48959543
Raw reads	243896508	204592452	253503938	192147514
Raw data yield (Mb)	21951	18413	22815	17293
Reads mapped to genome	204193470	145810600	202993882	156292035
Reads mapped to target region^(2)^	88581652	101995817	138982919	108872112
Data mapped to target region (Mb)	6298.25	7305.56	9902.46	7748.06
Mean depth of target region (X)	128.64	148.90	202.26	158.25
Coverage of target region (%)	98.77	98.56	98.81	98.75
Average read length (bp)	89.87	89.84	89.78	89.85
Total quality-passing SNPs	116950	114204	119780	117371
Total quality-passing indels	7442	7361	7773	7500

### Bioinformatic Analysis Identifies 22 Candidate Genes

In total, 82 variants shared by the 2 cases remained through the exclusion of 4 public genetic databases (the procedures are shown in Table 7), and no reported IBD single nucleotide variant was found. After performing filtering steps for gene function and mutation prediction, we obtained 22 candidate genes (Table 8). Using 4 internet tools, we acquired the top 6 genes from the 22 candidates: *THBS1*, *KLF4*, *SYNE1*, *CHD8*, *PDCL* and *DLG1*. These genes were the most likely to be the genetic cause of the 2 affected patients.

**Table 7:** Filtration of SNPs/Indels.

Individual ID	Grandmother	Mother	Daughter	Father
Total SNPs and indels	173991+7773	172848+7500	172005+7442	173055+7361
Quality-passing SNPs and indels	119780+7773	117371+7500	116950+7442	114204+7361
Protein-disrupting SNPs and indels (PDSI)	14553+1369	14345+1314	14581+1280	14547+1294
PDSI after filtering against dbSNP	2144+382	2138+366	2108+348	2129+359
PDSI after filtering against dbSNP+1000 Genomes	1459+220	1498+221	1469+189	1448+208
PDSI after filtering against dbSNP+1000 Genomes+HapMap	1457+220	1497+221	1467+189	1446+208
PDSI after filtering against dbSNP+1000 Genomes+HapMap+YH	1420+220	1460+221	1438+189	1413+208
PDSI after filtering against dbSNP+1000 Genomes+HapMap+YH+inhouse dataand fitting a dominant model **(shared by two cases)**	0	0	82+0	
Filtered candidate genes			**22**	
Sanger sequence for validation			**22**	

**Table 8:** List of 22 candidate genes and mutations prediction.

NO	Chromosome	Position	Reference	Gene name	Codons	SIFT Prediction	MutationTaster Prediction	ConsScore GERP
1	chr6	90418252	C	*MDN1*	GAC7861CAC	DAMAGING	polymorphism	2.23
2	chr14	21860964	C	*CHD8*	CGT5636CAT	DAMAGING	disease causing	5.34
3	chr9	125582872	T	*PDCL*	GAT398GGT	DAMAGING	disease causing	5.47
4	chr17	55183450	G	*AKAP1*	GTG625ATG	DAMAGING	polymorphism	4.22
5	chr15	39885853	G	*THBS1*	GGA3251GAA	DAMAGING	disease causing	5.78
6	chr16	16170185	G	*ABCC1*	GGG1915TGG	DAMAGING	disease causing	4.11
7	chr1	1470881	G	*TMEM240*	TCG380TTG	DAMAGING	disease causing	3.37
8	chr7	73279329	C	*WBSCR28*	CAG79AAG	DAMAGING	polymorphism	4.43
9	chr9	2717768	C	*KCNV2*	TCC29TGC	DAMAGING	disease causing	4.45
10	chr7	99160120	A	*ZNF655*	–	–	–	3.92
11	chr15	41275952	G	*INO80*	–	–	–	2.69
12	chr10	116225553	G	*ABLIM1*	CGG1345TGG	DAMAGING	disease causing	3.37
13	chr1	26368197	T	*SLC30A2*	ATG685GTG	DAMAGING	disease causing	5.6
14	chr20	30232655	T	*COX4I2*	GTG464GCG	DAMAGING	disease causing	4.38
15	chr6	167570520	G	*GPR31*	ACG800ATG	DAMAGING	polymorphism	2.65
16	chr6	168366533	G	*MLLT4*	GGA4993AGA	DAMAGING	disease causing	5.03
27	chr16	20492206	C	*ACSM2A*	ACG1472ATG	DAMAGING	polymorphism	3.26
18	chr20	420894	C	*TBC1D20*	GTG766ATG	DAMAGING	disease causing	5.65
19	chr9	110249480	G	*KLF4*	–	–	–	3.45
20	chr3	196921405	A	***DLG1***	ATC374ACC	TOLERATED	disease causing	5.17
21	chr6	152536152	C	*SYNE1*	CGT22022CAT	TOLERATED	disease causing	5.07
22	chr7	87051466	T	*ABCB*	ATT2287GTT	TOLERATED	polymorphism	4.85

### Sanger Sequencing and Genotyping Combined with Bioinformatic Analyses Identifies DLG1 as a Potential Susceptibility Gene

Sanger sequencing confirmed the presence of the 22 mutations in the affected father and daughter. 10 healthy members of family A were sequenced to test for these variants. We found that one family member carried the variant in the *KLF4* gene. The other 21 mutations absent in healthy family members showed co-segregation.

The genotyping of the 22 SNVs indicated that 8 variants in *THBS1*, *SYNE1*, *CHD8*, *TMEM240*, *AKAP1*, *COX4I2*, *ZNF655* and *KCNV2* were positive in 401 HC, whereas the other 14 variants were negative. We again focused on the 6 top candidate genes (*THBS1*, *KLF4*, *SYNE1*, *CHD8*, *PDCL* and *DLG1*) identified through the prioritization analysis. In contrast to *THBS1*, *KLF4*, *SYNE1* and *CHD8*, none of the 401 HC was found to carry *PDCL* or *DLG1* mutations. Subsequent genotyping of 22 SNVs in 401 sporadic IBD cases indicated that one female CD patient aged 21 years carried a mutation in *DLG1* (Table 9), and no patients had variation in *PDCL*. A PLINK analysis showed the variant in *DLG1*was of significance (*P*<0.05).

**Table 9:** Distributions of rare variants in the *DLG1* gene.

Patient ID	Gender	Age (years)	Nucleotidechange	Amino acidchange	ChromosomePosition	Exon	Sequencing method
**CD Family A**							
Father(diagnosed)	Male	44	c.374T>C	p.I125T	chr3_196921405	4	Exome
Daughter(diagnosed)	Female	16	c.374T>C	p.I125T	chr3_196921405	4	Exome
Mother(unaffected)	Female	42	–	–	–	–	Exome
Grandma(unaffected)	Female	81	–	–	–	–	Exome
**CD Family B**							
Case 1(diagnosed)	Male	39	c.833G>A	p.R278Q	chr3_196865242	9	Direct PCR sequencing
Case 2(diagnosed)	Male	42	c.833G>A	p.R278Q	chr3_196865242	9	Direct PCR sequencing
Case 3(diagnosed)	Female	32	c.833G>A	p.R278Q	chr3_196865242	9	Direct PCR sequencing
Case 4(diagnosed)	Female	24	c.833G>A	p.R278Q	chr3_196865242	9	Direct PCR sequencing
CJ 5(undiagnosed)	Female	56	c.833G>A	p.R278Q	chr3_196865242	9	Direct PCR sequencing
CJ 6(undiagnosed)	Female	6	c.833G>A	p.R278Q	chr3_196865242	9	Direct PCR sequencing
CJ 7(undiagnosed)	Female	6	c.833G>A	p.R278Q	chr3_196865242	9	Direct PCR sequencing
CJ 8(undiagnosed))	Male	7	c.833G>A	p.R278Q	chr3_196865242	9	Direct PCR sequencing

**Figure 2:**
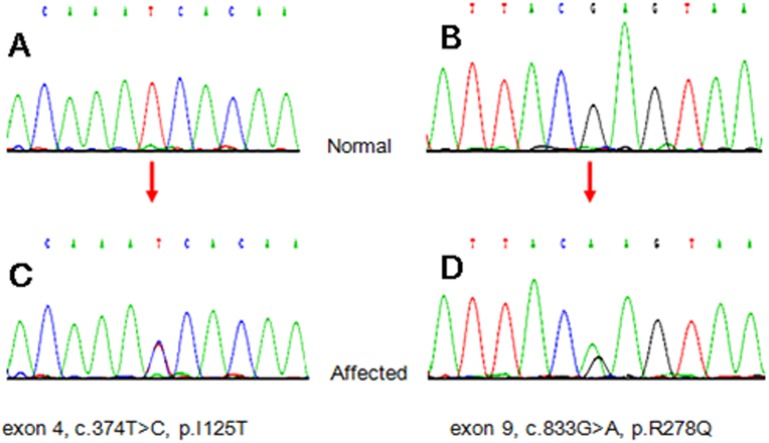
Chromatogram of *DLG1* gene mutations. The Sanger sequence traces from normal human controls are shown in panel A and B; the mutations were heterozygous at the corresponding locus (orange arrows indicating) in panel C and D.

By examining all of the exons of *PDCL* and *DLG1* in 25 young and intractable CD patients, we found two cases (Table 2, Patient ID are 3 and 4) who carried another variant in *DLG1* (Figure 2, exon 9, c.833G>A, p.R278Q). We traced Patient 3, 4 and their families, and found that two cousin sisters (Cases CJ2 and CJ3) and one brother (Case CJ4) of Patient 4 who were unexpectedly found to have ulcers in the terminal ileum by endoscopy, and a biopsy showed non-specific chronic inflammation. After being treated with 5-ASA and azathioprine, four affected cases in this family have almost achieved their colonic mucosal healing. Cases CJ2, CJ3 and CJ4 were all found to be carriers of mutation R278Q (c.833G>A) by Sanger sequencing, and the family was called family B. We found 4 unaffected carriers (CJ5, CJ6, CJ7 and CJ8) of this variant after sequencing the other 15 members of family B, and these individuals will be followed up. CJ5 received a diagnosis of rheumatic heart disease with arthritis. The variants and carriers of *DLG1* are listed in Table 9. Neither of the monozygotic CD twins carried any mutation in all 3 exons of *PDCL* or in all 25 exons of *DLG1*.

Bioinformatics analyses were used to dissect the two non-synonymous mutations of *DLG1* found in the study described above. MutationTaster showed that the variant in *DLG1* (Figure 2, c.374T>C, p.I125T) was likely to be disease-causing. We compared the SNV sequence of species at different evolutionary distances by GERP and found that the amino acid substitution of *DLG1* was highly conserved. Regarding another variant of *DLG1* (Figure 2, exon 9, c.833G>A, p.R278Q), the PMut analysis of the mutation indicated that it is pathological (http://mmb.pcb.ub.es/PMut/), and the prediction from PolyPhen-2 was that the mutation was most likely damaging; however, the MutationTaster analysis indicated polymorphism, and SIFT predicted the mutation to be tolerated.

## Discussion

Rare and low-frequency variants might have substantial effect sizes in complex disorders such as IBD [18]. A main goal of human genetic studies is to identify uncommon variants that play important roles in pathogenesis and reveal the familial transmission of diseases [6], [8]. Furthermore, uncommon alleles shared by affected individuals in a family are more prone to familial clustering of disease than common alleles carried in a population.

In this study, we applied whole exome sequencing to anatomize the genetic background of a Chinese family with CD and successfully identified genetic variants in the coding regions of the *DLG1* gene that may be associated with increased risk of CD. We first identified a novel SNV c.374T>C (p.I125T) in exon 4 of *DLG1* through whole exome sequencing and bioinformatic analysis. In subsequent validation studies, we also identified 4 CD patients of another Han Chinese family harbored the variant c.833G>A. Altogether these data suggest that *DLG1* is a susceptible gene for CD.

*DLG1* encodes a multi-domain scaffolding protein, which may have a role in septate junction formation, signal transduction, cell proliferation, synaptogenesis and lymphocyte activation (http://www.ncbi.nlm.nih.gov/gene/). The DLG1 protein is composed of an N-terminal L27b oligomerization domain, a proline-rich domain (PRD), three PDZ (PSD-95, Dlg and ZO-1) domains, an SH3 (Src Homology 3) domain and a catalytically inactive GUK (GUanylate Kinase) domain. During antigen recognition, these modular domains allow DLG1 to co-localize with synaptic actin, translocate into sphingolipid-rich microdomains within the IS and associate with Lck, ZAP-70, Vav, WASp Ezrin and p38 [19]. *DLG1* has been shown to play roles in T cell polarity and T cell receptor signal specificity [20], [21], and be involved in the generation of memory T cells [22]. The loss of *DLG1* leads to increased invasion in response to pro-tumorigenic cytokines, such as IL-6 and TNF-α [23], [24].

In accord with the suggested autoimmune nature of CD, strong evidence has implicated T cells and T-cell migration to the gut in initiating and perpetuating the intestinal inflammatory process and tissue destruction [25], [26]. Anti-cytokine agents are therefore likely to be useful in the treatment of IBD [27], [28]. After intravenous injection with six cycles of infliximab, the affected daughter in Family A has almost achieved mucosal healing of her colonic disease and was likely to have a better prognosis than those *DLG1* mutation carriers who did not accept infliximab treatments in our study. It was corroborative evidence that *DLG1* was causative for the CD patients of the two Chinese families.

Complex human disease is a large collection of individually rare, even private variants [29]. A single locus can harbor both common variants of weak effect and rare variants of strong effect [30]. The results of our study of two CD families indicated genetic heterogeneity and susceptibility. We analyzed family A using an autosomal dominant model, and several factors were important to the success of this study.

First, according to the database at our center [31], although the incidence of CD and UC is still low, the number of cases and severity of disease are increasing in China [32], [33], which provides the appropriate conditions to recruit patients for the subsequent validations.

Second, a stepwise approach was taken to help narrow down the list of genetic variants responsible for this disease. For the genetic susceptibility of CD, despite the success of GWAS in identifying significantly associated loci [34], the currently identified variants are estimated to account for less than a quarter of the predicted heritability [35]. Uncommon alleles may be maintained at a lower frequency in the population through negative selection, and it is not possible to create a complete catalog in the general population [36]. Therefore, rare causal variants are not likely to be found in public SNP databases and control exomes [37]. We did not find mutations in any reported susceptibility genes that were shared by the affected father and daughter, which suggested that other variants may be associated with CD in these 2 individuals. To predict the impact of nonsynonymous variants, we applied 4 popular methods (PolyPhen2, SIFT, MutationTaster and PMut) [38], [39]. However, none of these methods was perfectly sensitive or specific. Regarding the mutation c.374T>C, SIFT and MutationTaster predicted it to be tolerated and disease causing, respectively. Different prediction algorithms used different information, and each had its own relative merits. It is thought to be better to use predictions from multiple algorithms rather than relying on a single one [40], [41]. We also used several different bioinformatic methods to filter and prioritize the SNVs and genes to increase the robustness of the analysis results.

Finally, to confirm the results and identify the susceptibility gene, we used genotyping and Sanger sequencing methods for validation. Traditional Sanger sequencing is the gold standard for mutation detection [9]. We were able to narrow the scope to only a few genes through these steps. By scanning all exons of *DLG1* and *PDCL*, a nonsynonymous variant c.833G>A of *DLG1* was found in family B, thus confirming that *DLG1* is a gene whose mutation is associated with high risk.

Some limitations must be addressed. First, IBD patients with family history are rare among Han Chinese. In this family study, there were only two affected members, so the size of the pedigree was small. Second, the patients studied did not have an onset as early as those were previous reported in Caucasian population [42], [43]. Third, because of genetic heterogeneity, the variants appear to be present only in a subset of CD patients, and were not carried by the pair of monozygotic twins studied. Furthermore, in complex diseases, a central problem is that each variant only makes a small contribution to the disorder [44]. Other candidate genes discovered by us, such as *THBS1*, *KLF4*, *SYNE1*, *CHD8* and *PDCL*, may also contribute to CD. However, variation in these genes must be identified in more cases and controls. Additionally, considering that the variant was also present in the unaffected individuals of family B, other disease-causing factors lying outside of our set of candidate genes may also exist [45]. Finally, functional analyses are needed to elucidate the biological role of this gene in CD susceptibility.

In conclusion, we report the discovery of coding region variants in *DLG1* in human CD through whole exome sequencing and bioinformatic analysis and identify *DLG1* as a potential susceptibility gene for CD in the Chinese population. Our study also demonstrates that whole exome sequencing is an efficient and cost-effective genetic strategy. Bioinformatic approaches are likely to become useful tools for the discovery of genes and to provide important guidance for finding rare variants in a complex disorder. Finding different, rare and pathogenic mutations in the same gene in unrelated individuals with the same phenotype provides important support for our study. However, confirmation of *DLG1*’s involvement in CD pathogenesis still requires validation in further functional experiments and clinical trials. Personalized medicine is also anticipated to be developed based on definite biological processes and molecular causes.
